# SiYGL2 Is Involved in the Regulation of Leaf Senescence and Photosystem II Efficiency in *Setaria italica* (L.) P. Beauv.

**DOI:** 10.3389/fpls.2018.01308

**Published:** 2018-09-04

**Authors:** Shuo Zhang, Hui Zhi, Wen Li, Jianguo Shan, Chanjuan Tang, Guanqing Jia, Sha Tang, Xianmin Diao

**Affiliations:** Institute of Crop Sciences, Chinese Academy of Agricultural Sciences, Beijing, China

**Keywords:** EGY1, *Setaria italica*, chlorotic mutant, leaf senescence, PS II efficiency

## Abstract

A yellow-green leaf mutant was isolated from EMS-mutagenized lines of *Setaria italica* variety Yugu1. Map-based cloning revealed the mutant gene is a homolog of *Arabidopsis thaliana AtEGY1*. EGY1 (ethylene-dependent gravitropism-deficient and yellow-green 1) is an ATP-independent metalloprotease (MP) that is required for chloroplast development, photosystem protein accumulation, hypocotyl gravitropism, leaf senescence, and ABA signal response in *A. thaliana*. However, the function of EGY1 in monocotyledonous C_4_ plants has not yet been described. The *siygl2* mutant is phenotypically characterized by chlorotic organs, premature senescence, and damaged PS II function. Sequence comparisons of the AtEGY1 and SiYGL2 proteins reveals the potential for SiYGL2 to encode a partially functional protein. Phenotypic characterization and gene expression analysis suggested that SiYGL2 participates in the regulation of chlorophyll content, leaf senescence progression, and PS II function. Additionally, our research will contribute to further characterization of the mechanisms regulating leaf senescence and photosynthesis in *S. italica*, and in C_4_ plants in general.

## Introduction

AtEGY1 is an ATP-independent metalloprotease (MP) belonging to the M50 family (van der Hoorn, 2008). Members of the M50 family typically contain four to eight transmembrane helices (TMHs), three adjacent ones of which can form a conserved three-TMH core structure. The conversed motifs HEXXH and NPDG are located in the first TMH and the third TMH of the core structure, respectively (Kinch et al., 2006; Feng et al., 2007; Koussis et al., 2017). M50 family members contain three metal ligand-binding sites required for protease activity. Two of the ligand-binding sites reside in the HEXXH motif and the third is coordinated by the Asp residue in NPDG motif (Yu and Kroos, 2000; Feng et al., 2007). As MPs, M50 family proteins participate in multiple cellular processes by cleaving substrates such as membrane-tethered proteins, transcription factors, and signal peptides (Bölter et al., 2006; Kinch et al., 2006; Mukherjee et al., 2009; Che et al., 2010; Saito et al., 2011). AtEGY1 is targeted to chloroplasts and can be upregulated by both light and ethylene treatment in *A. thaliana* (Chen et al., 2005). The function of AtEGY1 has been characterized in a number of different studies. AtEGY1 has three conversed motifs: GNLR, HEXXH, and NPDG. The first is a signature motif unique to EGY family proteins (Chen et al., 2005); the function of this motif has not yet been clarified. The latter two motifs are metal-chelating motifs unique to M50 family members. AtEGY1 was shown to play a role in many biological processes including the regulation of chloroplast development, ethylene-dependent hypocotyl gravitropism, the accumulation of membrane-bound chlorophyll *a*/*b* binding (CAB) proteins, and responding to ammonium and phosphate stress (Chen et al., 2005; Guo et al., 2008; Li et al., 2012; Yu et al., 2016). In addition, AtEGY1 was reported to be involved in leaf senescence. The T-DNA insertion mutant of *AtEGY1, Ategy1*, had yellow rosette leaves and reduced chlorophyll content, leaf survival, and Fv/Fm. Mutants also showed decreased soluble protein content and increased ion leakage compared with WT. The expression of senescence-associated genes were increased in *Ategy1*. Under dark treatment, the aging phenotype in *Ategy1* was more obvious than that in WT. These data indicated that the loss of AtEGY1 function accelerated leaf aging and decreased photosystem II (PS II) efficiency. In addition, exogenously applied glucose could rescue these mutant phenotypes (Chen et al., 2016).

Leaf senescence is an important plant developmental process that is associated with a range of unique physiological and biochemical characteristics. During leaf senescence, leaves turn yellow; chlorophyll is degraded; proteins, fatty acids, nucleic acids, and other macromolecules are metabolized; plastids disintegrate rapidly; and nitrogen and nutrients are efficiently transferred into growing tissues and sink organs (Yang and Ohlrogge, 2009; Sakamoto and Takami, 2014; Diaz-Mendoza et al., 2016). Concurrently, thousands of senescence-associated genes are upregulated or downregulated at the transcriptional or post-transcriptional level as senescence is a well-controlled process that transfers nutrients from source to sink (Gupta et al., 2016; Wu et al., 2016). Crop yield and quality are, therefore, closely related to the timing of senescence. Proteins in old organs are extensively degraded into amino acids, amides, and ammoniums. For cereal crops, leaf senescence provides most of the nitrogen in grains. It has been reported that delayed senescence could lead to high yields (Lu et al., 2017) because the filling period is prolonged and sugar and nitrogen accumulation is increased (Egli, 2011; Gregersen et al., 2013). Conversely, grain quality has negative correlations with senescence progression. Because delayed senescence could cause inefficient nitrogen remobilization. The grain quality parameters such as proteins and micronutrients are diluted by carbohydrates, thereby leading to a low grain quality (Gregersen et al., 2008). There is a balance between grain yield and quality. Thus, the better understanding of the senescence process could help improve crop production and seed quality (Diaz-Mendoza et al., 2016).

Metalloproteases (MPs) are one type of plant proteases, which plays important roles in leaf senescence. There are approximately 100 MPs in plants, belonging to 19 families which are classified by the similarity in amino acid sequence. Although they are involved in many biological processes, the roles of MPs in leaf senescence are poorly understood (van der Hoorn, 2008). The most studied senescence-associated MPs are FtsH (filamentation temperature sensitive H) proteases that belong to the M41 family. FtsH1, 2, 5, and 8 can form a hexameric ring in the chloroplast thylakoid to regulate the thylakoid structure and remove photodamaged D1 protein in PS II under light stress (Yoshioka-Nishimura et al., 2014). Changes in the expression of *FtsH1, 2*, and *5* observed in senescing and aging leaves was found to be associated with light stress, dark conditions, or nitrogen stress (Roberts et al., 2012). FtsH6 is reportedly involved in detached leaf senescence and dark-induced leaf senescence. Other proteases including members of the M10 and M17 MP families have also been reported associated with leaf senescence. Matrix MPs (MMPs) belong to the M10 family and are reportedly upregulated during senescence (Roberts et al., 2012). A mutant of At2-MMP shows a late flowering and early senescence phenotype, suggesting that MMPs are responsible for senescence regulation (Golldack et al., 2002). Leucine aminopeptidase 2 (LAP2) is a member of the M17 family. In *A. thaliana*, a mutant of LAP2 displays an early-senescence phenotype, suggesting that LAPs are involved in leaf longevity (Waditee-Sirisattha et al., 2011). EGY1 belongs to M50 family. To date, seven M50 family members have been identified in *A. thaliana, Oryza sativa*, and *Zea mays*. These proteases play roles in chloroplast development, hypocotyl elongation and gravitropism, stress response, nuclear plastid signaling pathway, and plant development, respectively (Nishimura et al., 2016).

*Setaria italica* is a new model C_4_ monocotyledonous species that promises to accelerate functional genomics studies in the grasses (Doust et al., 2009). In this study, a pale green mutant *siygl2* was isolated from EMS-mutagenized lines of the *S. italica* Yugu1 cultivar. Map-based cloning indicated that the mutant gene encoded SiYGL2, a homolog of AtEGY1. Phenotypic surveys showed that *siygl2*, such as *Ategy1*, is characterized by chlorotic organs, premature senescence, and damaged PS II function. Unlike *Ategy1*, however, chloroplast development is not impaired in *siygl2*. Our study focused on the function of SiYGL1 in regulating senescence and photosynthesis in *S. italica* to supplement the existing functional gene knowledge available for this C_4_ model plant. Additionally, our research provides further insight into the mechanisms underlying the regulation of leaf senescence.

## Materials and Methods

### Plant Materials and Growth Condition

The *siygl2* mutant was identified in screens of EMS mutagenized populations of *S. italica* cultivar Yugu1. To determine the chlorophyll content and the photosynthetic rate, plants were planted under natural conditions in the experimental field of institute of Crop Sciences, Chinese Academy of Agricultural Sciences, in Beijing (116.6°E, 40.1°N) in summer season. For dark-induced senescence, detached leaves were incubated in water at 28°C.

### Chlorophyll Content, Photosynthetic Rate, and Chlorophyll Fluorescence

For analysis of photosynthetic pigments, leaves were cut into pieces and soaked in 95% alcohol for approximately 72 h until leaf pieces were completely transparent. The absorbance values of the supernatant were measured at 665 and 649 nm with UV-1800 ultraviolet/visible light. Chlorophyll *a* (Chl *a*) and chlorophyll *b* (Chl *b*) levels were then calculated as described by Lichtenthaler (1987). Statistics analysis was conducted using Welch’s two-sample *t* test. Multiple comparisons were made with LSD by IBM SPSS Statistics 23.0. The photosynthetic rate and chlorophyll fluorescence were measured on sunny days using a Li-6400 portable photosynthesis system (LI-COR, Lincoln, NE, United States) using mature leaves from five individuals. The light source used for measuring the photosynthetic parameters is 6400-02B LED light source, the ParIn parameter was set to 1000 μmol m^-2^ s^1^. The equations for the photosynthetic parameters are calculated as follows (Hu et al., 2014).

ΔΦPS II=(Fm′- Fs)/Fm′                                      ΔETR=PAR (photosynthetic active radiation)× ΔΦPS II × 0.84 × 0.5

### Transmission Electron Microscopy

The second leaf and seventh leaf were obtained from plants that were at the eight-leaf stage. Leaf material was cut into 2 mm × 1 mm pieces and fixed overnight in 0.1 M phosphate buffer with 2.5% glutaraldehyde. The samples were then washed with 0.2 M phosphate buffer three times and post-fixed in 1% osmium tetroxide for 1 h. After staining with uranyl acetate, samples were further dehydrated in a gradient ethanol series and finally embedded into resin. Ultrathin sections were made and examined by JEM 1230 transmission electron microscopy (TEM). The areas of plastoglobulis are calculated by Image-Pro plus 6.0 (Media Cybernetics, Silver Spring, Georgia Avenue, United States). Statistics treatment was made with Welch’s two-sample *t* test.

### Map-Based Cloning

An F2 population generated from a cross between *siygl2* and the SSR41 cultivar was used for mapping of the *SiYGL2* locus. Sixty-five SSR markers based on earlier studies (Jia et al., 2009; Zhang et al., 2014) were adopted for gene cross positioning. Thirteen cleaved amplified polymorphism sequences (CAPS) markers were newly designed for fine mapping based on the single-nucleotide polymorphism information between Yugu1 and SSR41 (Jia et al., 2013) (**Supplementary Table S1**). To identify mutant locations, 40 sequencing primers were developed that covered the whole candidate region based on the genome sequence information from the *S. italica* genome project V2.2^1^ database (**Supplementary Table S2**).

### RNA Preparation and Transcript Analysis

RNA was isolated from wild-type (WT) and *siygl2* stems, panicles, and 1st and 10th leaves from the top of the plant at the heading stage from fresh plant tissues using a Pure Link RNA Mini Kit (Cat no. 12183018, Invitrogen, United Kingdom). First-srtand cDNA was synthesized with a PrimerScript 1st Strand cDNA Synthesis Kit (Cat no. 6210A, TaKaRa, Otsu Shiga, Japan). Quantitative PCR was conducted using a Fast Start Universal SYBR Green Master (ROX) (Cat no. 04913914001, Roche, Mannheim, Germany) using the specific primers listed in **Supplementary Table S3**. *Cullin* was selected as the reference gene according to a previous study (Martins et al., 2016). The data were detected and analyzed using an Applied Biosystems 7300 Analyzer (Applied Biosystems, Foster City, CA, United States). Statistics treatment was made with Welch’s two-sample *t* test.

### Bioinformatics Analysis

The sequences and structures of the candidate genes were obtained from the *S. italica* genome project V2.2. For phylogenetic analysis, homologs were obtained by NCBI Protein Blast^2^. Sequence alignments and cladograms were produced using MEGA 5.0 software. Protein TMHs were calculated using TMHMM_v.2^3^.

### Subcellular Localization

The full-length cDNA of *SiYGL2*, excluding the stop code, was amplified from Yugu1 using the following primers: 5′TATCTCTAGAGGATCCCTATCCTCCTTCGGTCCTTCCCATT 3′ and 5′ TGCTCACCATGGATCCGAACGAAGTAACAAGCCCTACACCT 3′ [the underlined sequences are adaptors for In-fusion^®^ PCR cloning system (Cat no. 072012, Clontech, United States) and contain *Bam*HI cleavage sites]. The cDNA sequences were cloned into the p16318hGFP vector to form fusion proteins with the C-terminus of GFP. These vectors were then transfected into foxtail millet protoplasts by PEG-mediated transformation and detected by confocal microscopy (LSM700, Carl Zeiss, Germany).

## Results

### The Chlorotic Mutant *siygl2* Has Reduced Chlorophyll Accumulation and Poor Agronomic Traits

**FIGURE 1:**
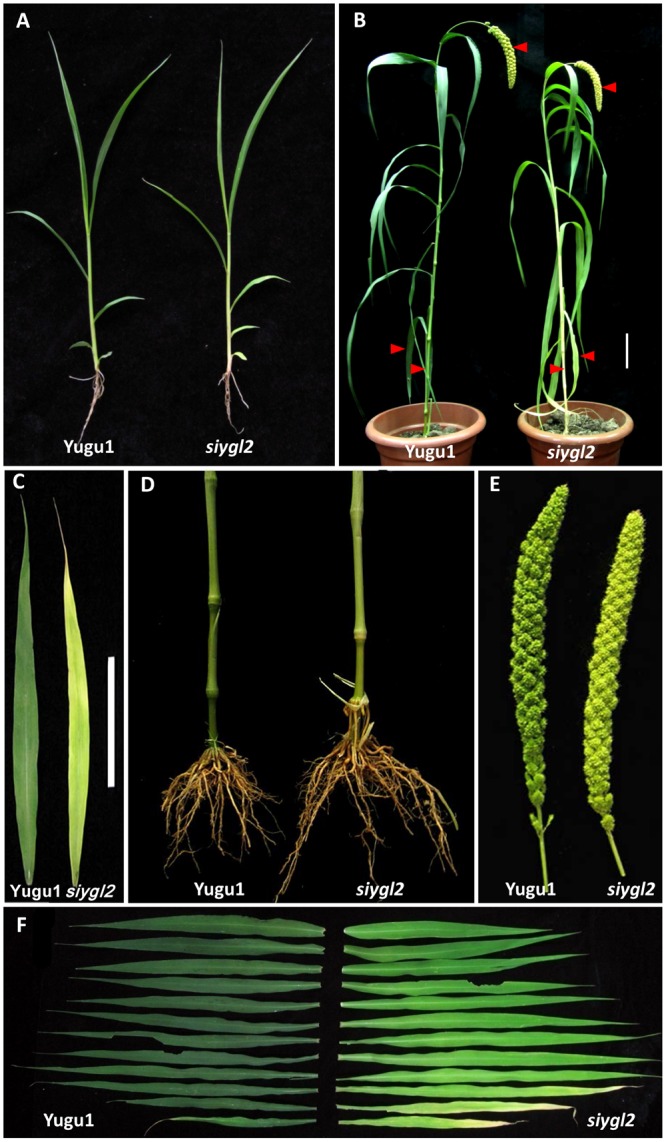
Phenotypic characteristics of the *siygl2* mutant. **(A)** Five-week-old seedling phenotypes of the wild-type (WT) cultivar Yugu1 and the *siygl2* mutant. **(B)** Heading stage phenotypes of Yugu1 and *siygl2.* The red bars point out the significant abnormal phenotypes in panicles, stems, and bottom leaves. **(C–E)** Lower leaves, stems, and panicles of heading stage plants. **(F)** Leaf color comparison of the WT (left) and *siygl2* (right) at the heading stage. Leaves from the top of the plant to the bottom are arranged accordingly.

The *S. italica* chlorotic mutant *siygl2* was generated from the Yugu1 cultivar by EMS treatment. The *siygl2* mutant showed a relatively normal phenotype in the seedling stage (**Figure 1A**). Throughout development, however, these plants gradually became chlorotic, and in the late developmental stages, *siygl2* produced chlorotic leaves, stems, and panicles (**Figures 1B–E**). Additionally, the lower leaves of *siygl2* had more abnormal phenotypes than the upper leaves (**Figure 1F**). Senescence appeared to be accelerated in *siygl2* as the basal leaves of *siygl2* were tip burned. Several key agronomic traits of *siygl2* and WT plants were analyzed. The results showed that some yield characteristics, such as floret grain number and panicle weight, are significantly reduced compared to WT (**Table 1**).

**Table 1:** Agronomic traits in wild-type Yugu1 and mutant *siygl2* plants.

Trait		Yugu1	*siygl2*	*P*-value
Plant height (cm)		139.6 ± 7.8	136.1 ± 5.7	0.117
Tiller number		2.8 ± 1.1	2 ± 0.7	0.212
Main panicle length (cm)	^∗^	18.4 ± 1.3	15.2 ± 1.8	0.013
Main panicle diameter (cm)	^∗^	28.8 ± 1.5	17.8 ± 6.4	0.021
Floret number		109.2 ± 13.2	124.6 ± 22.4	0.222
Floret grain number	^∗∗^	94.6 ± 26.1	41.6 ± 18.3	0.006
Panicle weight per plant (g)	^∗∗^	30.2 ± 9.5	10.8 ± 4.0	0.003
Main panicle weight (g)	^∗∗^	17.8 ± 1.4	9.0 ± 3.2	0.001
Thousand seed weight (g)		2.6 ± 0.1	2.7 ± 0.2	0.309

*Means and standard deviations were obtained from ten independent leaf samples grown under normal growth conditions. Means and standard deviations are obtained from 10 independent samples. Statistics treatment was made with Welch’s two-sample *t* test. ^∗∗^Significantly different at *P* = 0.01. ^∗^Significantly different at *P* = 0.05.*

### Analysis of the Chlorophyll Content and Photosynthetic Rate

**FIGURE 2:**
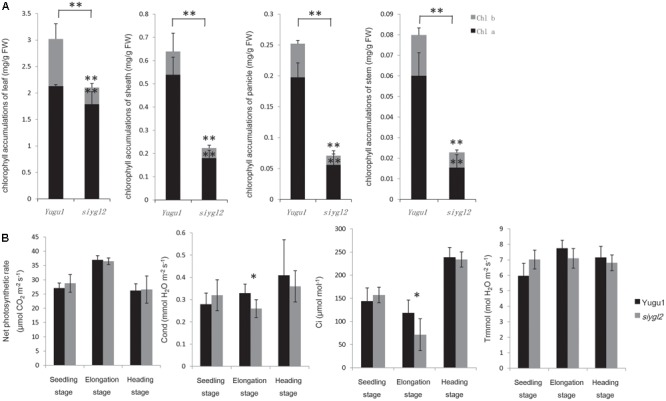
**(A)** Chlorophyll content of leaves, sheaths, panicles, and stems at the heading stage of wild-type cultivar Yugu1 and the *siygl2* mutant. Black and gray boxes show the amount of chlorophyll *a* and chlorophyll *b*, respectively. FW, fresh weight; Chl *a*, chlorophyll *a*; Chl *b*, chlorophyll *b*. Means and standard deviations were obtained from five independent leaf samples grown under normal growth conditions. **(B)** Photosynthetic parameters in different developmental stages. Means and standard deviations were obtained from five independent leaf samples grown under normal growth conditions. Photo, photosynthetic rate; Cond, stomatal conductance; Ci, intercellular carbon dioxide concentration; Trmmol, transpiration rate. Means and standard deviations are obtained from five independent samples. ^∗^Significantly different at *P* = 0.05. ^∗∗^Significantly different at *P* = 0.01.

To further characterize the chlorotic phenotype of the *siygl2* mutant, we measured the chlorophyll content of leaf, sheath, panicle, and stem at the heading stage (**Figure 2A**), and found them to have 71, 35, 28, and 28% the chlorophyll content of WT tissues, respectively. Both Chl *a* and Chl *b* levels were reduced in *siygl2*, with the reduction in Chl *b* being more severe in leaves.

As reductions in chlorophyll may be associated with changes of the photosynthetic capacity, the photosynthetic parameters of the newly emerging leaves (from the top of the plant) were measured (**Figure 2B**). Surprisingly, *siygl2* photosynthetic capacity was not significantly affected at the seedling stage, the elongation stage, or the heading stage. The stomatal conductance of *siygl2* decreased about 22% in the elongation stage concurrent with a decrease in the intercellular carbon dioxide concentration of approximately 40%. Overall, the decrease in chlorophyll accumulation in *siygl2* did not significantly affect photosynthesis in the newly developed leaves. Variations in the intercellular carbon dioxide concentration and stomatal conductance may be caused by other effects of the mutant gene or environmental factors. Further study in investigating the ACi curve of both WT and mutants would help to reveal the effects of EGY1 in photosynthesis.

### SiYGL2 May Be Involved in the Regulation of Leaf Senescence and PS II Efficiency

**FIGURE 3:**
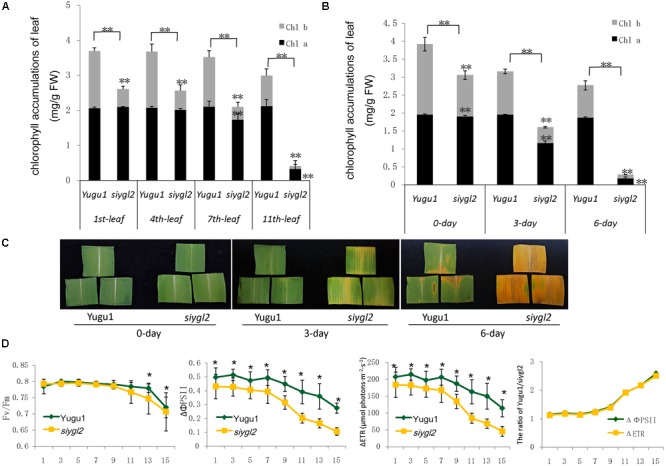
**(A)** Chlorophyll accumulation in different leaves at the heading stage (mg/g FW). Black and gray boxes represent Chl *a* and Chl *b*, respectively. **(B)** Chlorophyll accumulations after different durations of dark treatment. **(C)** Leaves of Yugu1 and *siygl2* after different durations of dark treatment. **(D)** Chlorophyll fluorescence in different leaves of Yugu1 and *siygl2*. The *X* axis shows the leaf position from the top of plants. For measuring the chlorophyll content and chlorophyll fluorescence, means and standard deviations are obtained from five independent plants for each genotype. ^∗^Significantly different at *P* = 0.05. ^∗∗^Significantly different at *P* = 0.01.

As the basal leaves of *siygl2* had a more obvious chlorotic phenotype than the top leaves and showed accelerated senescence (**Figures 1B,F**), the chlorophyll pigment levels of the 1st, 4th, 7th, and 11th leaves from the top of *siygl2* and WT Yugu1 plants were examined (**Figure 3A**). Chlorophyll accumulation in the first leaves of *siygl2* declined by approximately 30% compared with Yugu1. However, in basal leaves, *siygl2* only contained one-third of the chlorophyll of the WT leaves at the same position. This suggests that chlorophyll levels decline earlier in the old leaves of *siygl2* than in Yugu1. We also noticed that in the first and fourth leaves of *siygl2*, the Chl *b* levels decreased while Chl *a* levels did not. In the 11th leaves, both Chl *a* and Chl *b* in *siygl2* are sharply decreased compared with Yugu1 (**Figure 3A**). Anyhow, the accelerated leaf senescence of *siygl2* could be associated with changes in the function of SiYGL2.

To verify the changes in leaf aging in *siygl2*, we first conducted a detached leaf senescence assay using dark treatments (**Figures 3B,C**). After 3 days of dark treatment, first leaves of WT and *siygl2* were compared. The leaves of *siygl2* began turning yellow, while the leaves of the WT remained green (**Figure 3C**). After 6 d of dark treatment, *siygl2* leaves were completely yellow, whereas only the cut ends of the WT leaves showed yellowing (**Figure 3C**). The chlorophyll content also reflected the changes induced by dark treatment (**Figure 3B**). These results suggest that SiYGL2 helps to negatively regulate dark-induced leaf senescence, with senescence promoted by the loss of SiYGL2 function. In addition, we noticed that at the beginning of dark treatment, the effect on the reduction of Chl *b* levels are stronger than on the reduction of Chl *a* levels (**Figure 3B**). This is similar with the phenomenon showed in **Figure 3A**.

The maximum photochemical efficiency (Fv/Fm) is a senescence-associated index. We measured the Fv/Fm values of the 1st, 3rd, 5th, 7th, 11th, 13th, and 15th leaves of WT and *siygl2* plants. The Fv/Fm values of the WT Yugu1 plants decreased gradually from the upper to the lower leaves. The Fv/Fm values of the mutant *siygl2* leaves displayed a similar decreasing tendency, with levels declining more sharply than in the WT in the 13th and 15th leaves (**Figure 3D**). This suggests that the basal leaves of *siygl2* enter senescence earlier than Yugu1. We further tested effective PSII quantum yield (ΔΦPS II) and photosynthetic electron transport rate (ΔETR) to describe the changes PS II light-use efficiency (**Figure 3D**). Both ΔΦPS II and ΔETR declined generally in leaves when moving from the top to the bottom of the plant, indicating that PS II photosynthesis capacity decreases with aging. In the whole-plant leaves, both ΔΦPS II and ΔETR of *siygl2* were lower than in Yugu1. From the 11th leaf, the ratio of both ΔΦPS II and ΔETR of Yugu1/*siygl2* significantly increased compared with that in the upper leaves, indicating that these two indices drop more acutely than in *siygl2* (**Figure 3D**). These results verify that PS II photosynthesis capacity is decreased and senescence is premature in *siygl2*.

**FIGURE 4:**
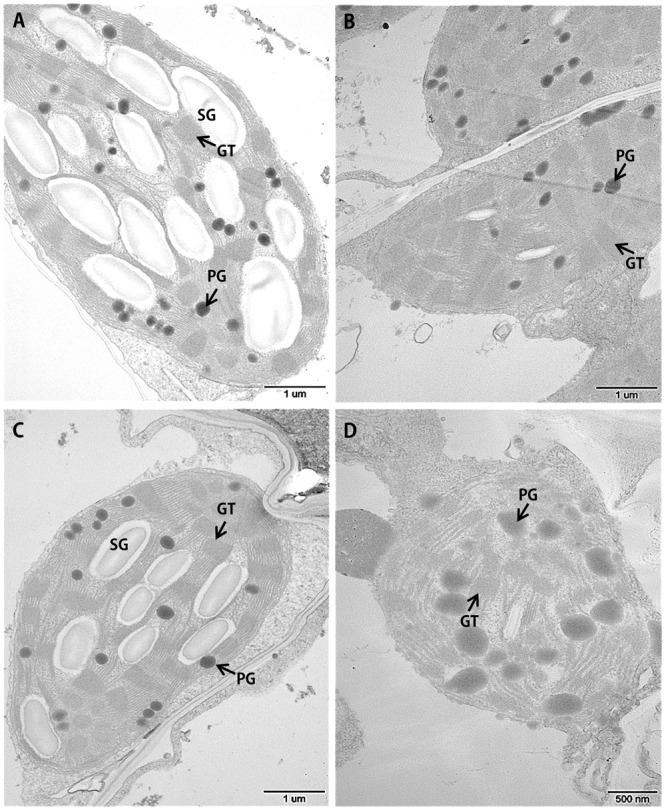
Transmission electron microscopic images of chloroplasts in the WT Yugu1 cultivar **(A,B)** and *siygl2* mutant **(C,D)**. **(A,C)** Chloroplasts in tender leaves. **(B,D)** Chloroplasts in tender aging leaves. SG, starch granules; PG, plastoglobuli; GT, grana thylakoid stacks.

Leaf senescence is always accompanied by changes in chloroplast ultrastructure. We obtained entirely expanded young leaves and basal leaves of plants at the elongating stage (eight leaf-stage) and surveyed the mesophyll cell-chloroplasts of these leaves. TEM observations showed that in the young leaves of *siygl2*, the chloroplasts were similar to those of Yugu1 (**Figures 4A,C**), indicating that thylakoid and chloroplast development were not impaired. However, in the basal leaves of *siygl2*, the chloroplasts were seriously disintegrated. The size of the plastoglobuli increased by 57.4% in *siygl2* (**Supplementary Figure S1**), and irregular grana thylakoid stacks were also be observed in the chloroplasts of the mutant (**Figure 4D**). In *siygl2* leaves at the same position as in Yugu1, chloroplasts retained their normal state (**Figure 4B**). This result indicates that chloroplast degradation of *siygl2* is advanced.

Taken together, the above results suggest that the *SiYGL2* gene may play a role in maintaining chlorophyll accumulation and chloroplast structure in aging leaves, and may delay the onset of leaf senescence. Suppression of SiYGL2 function could, therefore, lead to early senescence of basal leaves.

### Map-Based Cloning of the *SiYGL2* Locus and Bioinformatic Analysis

For genetic analysis of the *siygl2* mutant, we constructed an F_2_ population by hybridizing *siygl2* with the Yugu1 cultivar. The F_2_ progeny showed a segregation ratio of 3:1 (266:80, χ^2^ = 0.56 < χ^2^_0.05_ = 3.84), suggesting that this chlorotic phenotype was controlled by a single recessive gene.

**FIGURE 5:**
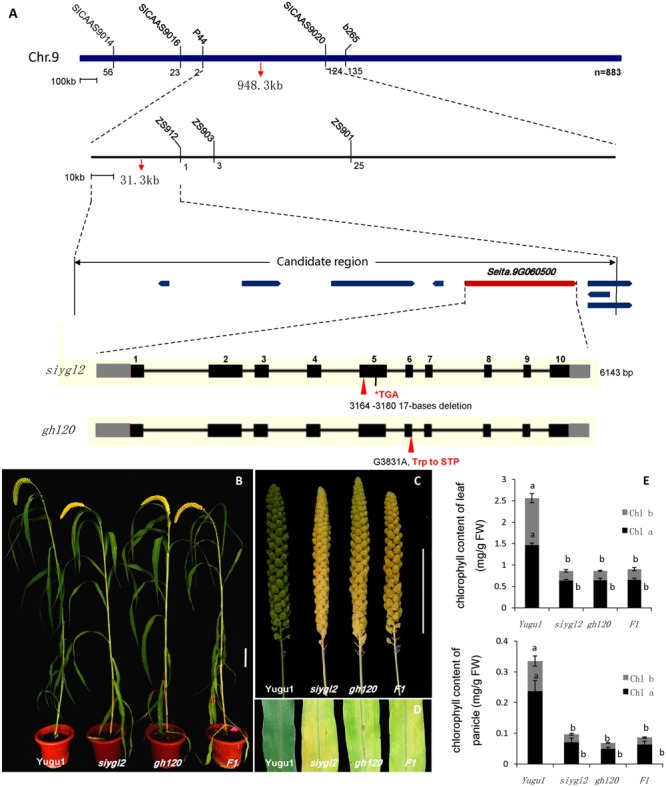
**(A)** Map-based cloning, gene structure of *Seita.9G060500*, and the mutant positions of *siygl2* and *gh120.* **(B–D)** are phenotypes of WT Yugu1 plants and mutants. **(E)** Chlorophyll content of *siygl2, gh120*, and F1 individuals. Means and standard deviations are obtained from five independent samples. Values connected by same letters are not statistically different; values not connected by same letters are statistically different at *P* = 0.05.

To map the *siygl2* locus, an F_2_ mapping population was generated from a cross between *siygl2* and the cultivar SSR41. Using this F_2_ population (883 yellow leaf plants), we generated a DNA pool of 40 individual mutant plants and preliminarily mapped the *siygl2* gene to a 948.3-kb genomic region on chromosome 9 by bulked segregation analysis. On this basis, a 31.3-kb genomic region between the SSR marker P44 and the CAPS marker zs912 was then defined. Within this region, eight open reading frames (ORFs) were predicted from the data on *phytozome*^4^ (**Figure 5A**). These ORFs were amplified and sequenced and only the fifth ORF (*Seita.9G060500*) was found to carry a 17-bp deletion (AATGTTTGACATATCAA) at the position 3,478,889–3,478,905 of chromosome 9. The gene structure of *Seita.9G060500* is predicted to contain 11 exons and 10 introns. The identified deletion in this gene leads to a frameshift that results in a termination codon at the fifth exon (**Figure 5A**).

We also identified another leaf chlorotic mutant, *gh120*, that shows the same phenotypes as *siygl2* (**Figures 5B–E**). Genome sequencing indicated that there is a single base change (G 3831 A) at the sixth exon of *Seita.9G060500* that leads to the alteration of Trp to a termination codon. The F_1_ individuals of a cross between *siygl2* and *gh120* are hemizygous at both the mutation sites of their parents (**Supplementary Figure S2**) and display similar phenotypes to their parents (**Figures 5B,D**). The chlorophyll content of F_1_ plants is consistent with that of *siygl2* and *gh120* mutant (**Figure 5E**). These results suggest that the mutations in *Seita.9G060500* indeed cause the abnormal phenotype in the *siygl2* and *gh120*.

**FIGURE 6:**
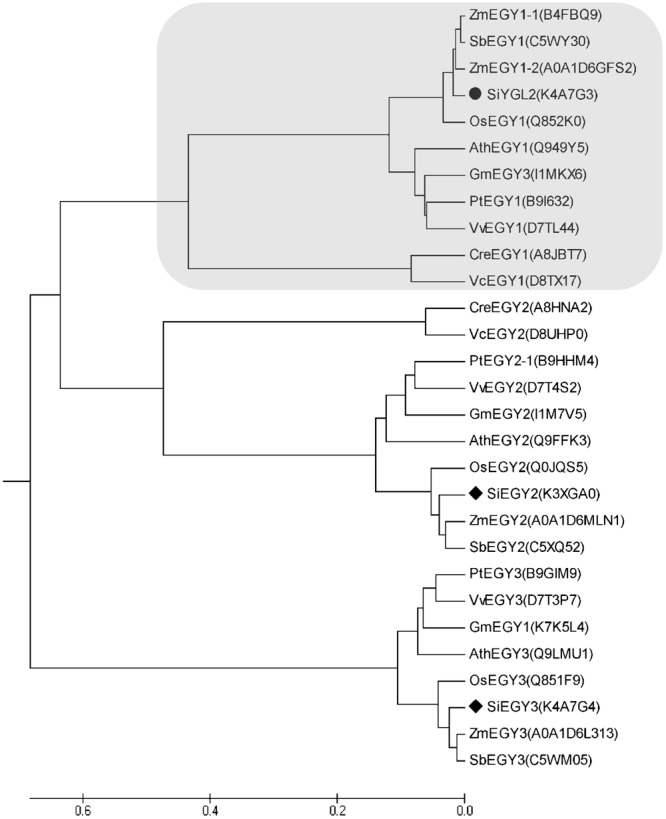
Phylogenetic analysis of EGY proteins in diverse species. Ath, *A. thaliana*; Cre, *Chlamydomonas reinhardtii*; Gm, *Glycine max*; Os, *Oryza sativa*; Pt, *Populus trichocarpa*; Sb, *Sorghum bicolor*; Si, *S. italica*; Vv, *Vitis vinifera*; Zm, Zea mays. Protein IDs are listed in brackets and are archived in the UNIPROT database (http://www.uniprot.org/). SiEGY2, Seita.5G097600. SiEGY3, Seita.9G108100.

Amino acid sequence comparison indicated that SiYGL2 is most closely related to AtEGY1, with these proteins sharing 77.3% amino acid sequence identity. SiYGL2 was, therefore, proposed to be a homolog of AtEGY1, a S2P-like chloroplast membrane-located MP. Phylogenetic analysis further confirmed this relationship (**Figure 6**). In *Arabidopsis*, there are three EGY proteins: AtEGY1, AtEGY2, and AtEGY3 (Chen et al., 2005). Similarly, two homologs of SiYGL2, Seita.5G097600 (SiEGY2) and Seita.9G108100 (SiEGY3), were identified in *S. italica* (**Figure 6**).

**FIGURE 7:**
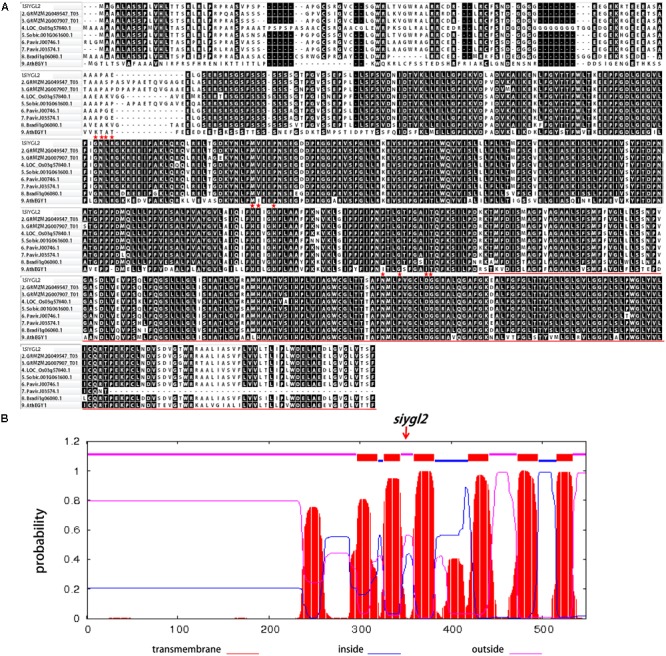
**(A)** Amino acid sequence alignment of the homologous proteins. Red stars show the position of the conserved motifs GNLR, HEXXH, and NPDG, respectively. The red lines show lost amino acid sequence of ΔSiYGL2. **(B)** Hydropathy plot of SiYGL2 and ΔSiYGL2. Red boxes show the transmembrane helices. Blue lines and pink lines at the top of the figure indicate the inside and outside peptide chains, respectively. The arrow indicates the mutated position of *siygl2.*

SiYGL2 is predicted to encode a 548-aa protein, with a peptide chain that contains three conversed motifs, GNLR (aa 169–178), HEXXH (aa 311–315), and NPDG (aa 442–454) (**Figure 7A**), that reportedly also occur in AtEGY1 (Chen et al., 2005). However, the mutant SiYGL2 protein (ΔSiYGL2) was predicted to lack 165 amino acid residues from the carboxyl terminus. Furthermore, frameshift mutation caused changes in the sequence from 362–383 aa, leading to the loss of NPDG motif. This suggests that the protein structure and function of ΔSiYGL2 is likely to differ significantly from that of SiYGL2.

Hydropathy analysis revealed that the AtEGY1 protein is highly hydrophobic as it contains six predicted transmembrane (tm) helices in its C-terminus (tm1, aa 340–362; tm2, aa 369–387; tm3, aa 402–424; tm4, aa 462–484; tm5, aa 516–538; tm6, aa 559–576). Conversely, ΔSiYGL2 lost four of these tm helices (**Figure 7B**).

### Expression Analysis of *SiYGL2*

**FIGURE 8:**
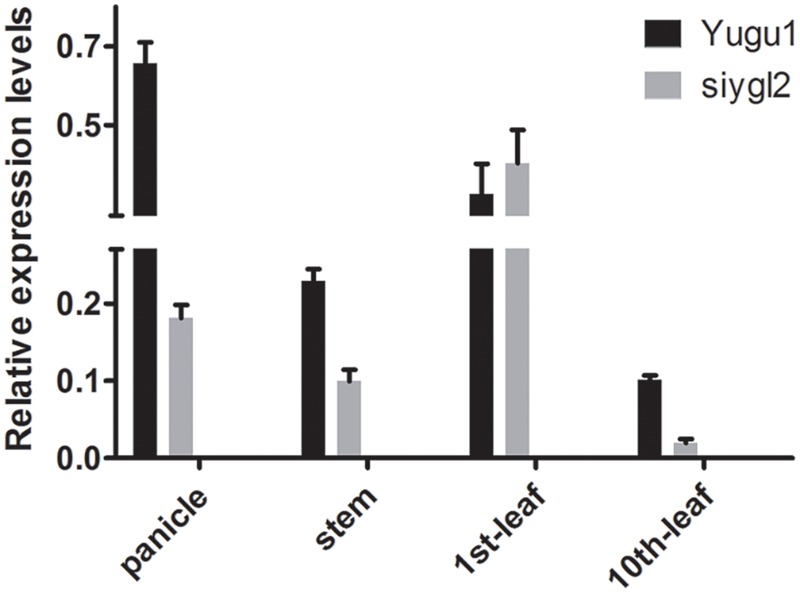
Transcript accumulation of *SiYGL2* in panicle, stem, and leaves. Black boxes, Yugu1; gray boxes, *siygl2*. Means and standard deviations are obtained from three independent samples and three independent assays. Statistics treatment was made with Welch’s two-sample *t* test. ^∗^Significantly different at *P* = 0.05.

As the *siygl2* mutant showed chlorotic leaves, stems, and panicles, with this phenotype more apparent in older leaves, the expression pattern of *SiYGL2* was investigated in different organs and leaves at different developmental stages by qRT-PCR (**Figure 8**). *SiYGL2* was more highly expressed in panicles and young leaves (1st leaves) than in stems and old leaves (10th leaves), suggesting that SiYGL2 is typically more active in young organs. However, *SiYGL2* expression in *siygl2* organs differed significantly; in panicle, stem, and the 10th leaf in *siygl2, SiYGL2* expression decreased to 27.7, 43.5, and 20.0% of those in WT, respectively. In the 10th leaf, in particular, transcript accumulation was very low. However, the expression *SiYGL2* in the first leaf of *siygl2* was higher (1.25-fold) than in the WT. These results suggest that the expression of *SiYGL2* in *siygl2* is not dependent on the function of the SiYGL2 protein.

### Subcellular Location Analysis of SiYGL2 Protein

**FIGURE 9:**
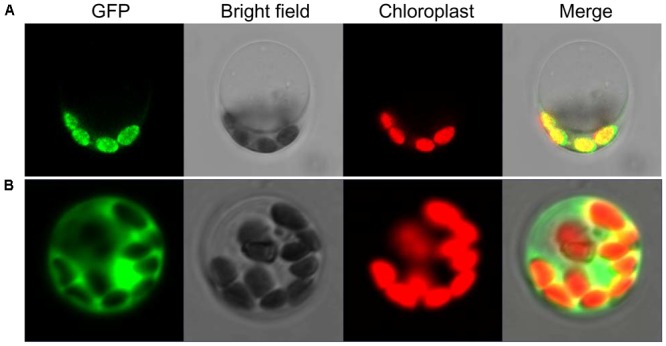
Subcellular localization of SiYGL2. **(A)** SiYGL2:GFP signal. **(B)** Empty GFP vector as a control.

To explore the subcellular location of SiYGL2, we generated a *SiYGL2-GFP* fusion gene. The fusion gene was placed under the control of the CaMV 35S promoter and introduced into protoplasts of Yugu1. The results obtained from confocal laser microscopy show that SiYGL2 is localized to the chloroplast (**Figure 9A**). This result is similar to that of AtEGY1 (Chen et al., 2005). In the GFP transgenic control line, GFP fluorescence was found in the cytomembrane, the plasma, and the nucleus (**Figure 9B**).

### Complicated Regulation of Senescence-Related Genes in the *siygl2* Mutant

**FIGURE 10:**
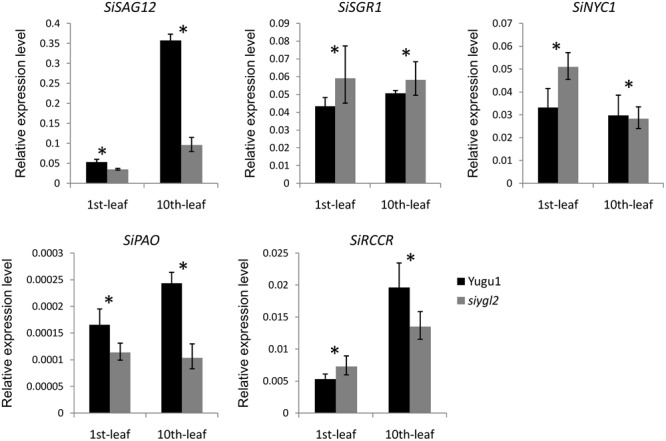
Relative expression levels of chlorophyll degradation- and leaf senescence-associated genes.

To further verify the influence of the *SiYGL2* mutation on leaf senescence, several senescence-related genes examined using qRT-PCR (**Figure 10**). *AtSAG12*, encoding a putative cysteine protease, is a negative regulator of senescence. In *A. thaliana*, expression of *AtSAG12* is strictly associated with senescence progression, with expression levels increasing throughout leaf aging before decreasing at the end of the aging process (Weaver et al., 1998). We used *SiSAG12*, a homolog of *AtSAG12* as a reporter to follow the progression of leaf senescence. In old 10th leaves of Yugu1, the expression level of *SiSAG12* was upregulated dramatically (approximately sevenfold), consistent with the reported expression of AtSAG12. While *SiSAG12* expression levels in both young and old leaves of *siygl2* were lower than those in the corresponding leaves of Yugu1. *SiSAG12* expression did increase by approximately threefold in 10th leaf than that in young leaf of *siygl2* (**Figure 10**). These results suggest that the expression of senescence-associated genes in *siygl2* is impaired, reflecting the variations in aging processes between *siygl2* and WT.

SGR1 and NYC1 are reportedly involved in chlorophyll degradation and disassembly of the light-harvesting complex of PS II (LHC II) during leaf senescence (Kusaba et al., 2007; Sakuraba et al., 2012). In the young leaves of *siygl2*, the expression of these two genes was higher than in the young WT leaves. Furthermore, the transcript accumulation of these two genes in mutant old leaves also differed significantly from their expression in WT. These results indicate that chlorophyll metabolism and LHC II stability during the aging process may be affected in the mutant. Two other senescence-associated genes, RCCR and PAO, also reportedly participate in chlorophyll degradation (Sakuraba et al., 2016). These two genes were upregulated in old WT leaves. In *siygl2*, however, the expression of these two genes differed significantly from the WT in both the 1st and 10th leaves. This suggests that chlorophyll degradation in *siygl2* is affected.

In summary, we conclude that changes in SiYGL2 function affects the regulation of several senescence-associated genes, with chlorophyll degradation and leaf senescence also affected.

## Discussion

### SiYGL2 Is Associated With the Regulation of Leaf Senescence, Chlorophyll Metabolism, and PS II Efficiency

Our study has shown that *SiYGL2* gene expression levels are higher in panicles and first leaves than in stems and old leaves (**Figure 8**). The flag leaf and panicle are the most important source and sink organs, respectively, and should retain high chlorophyll levels and photosynthetic capacities to avoid premature aging and ensure their continued output. The high expression of *SiYGL2* in these organs suggests that *SiYGL2* may play a role in maintaining the physiological state of the leaf and its photosynthetic capacity. Mutation of *SiYGL2* results in premature chlorophyll degradation, chloroplast disintegration, and leaf senescence in the basal leaves (**Figures 3A, 4**). Furthermore, aging of the leaves of the *siygl2* mutant can be induced more easily by dark than in the WT (**Figures 3B,C**). Taken together, these results suggest that SiYGL2 may be involved in delaying the onset of leaf senescence. This is consistent with the results reported for the SiYGL2 homolog AtEGY1 in *Arabidopsis* (Chen et al., 2016).

The chlorophyll content of *siygl2* was lower than in WT in both the top and basal leaves (**Figure 3A**), indicating that SiYGL2 may be related to chlorophyll metabolism. The chlorophyll degradation-associated genes SiNYC1, SiSGR1, and SiRCCR were expressed more highly in young leaves of *siygl2* than in Yugu1 (**Figure 10**), suggesting that chlorophyll degradation is mobilized early in young *siygl2* leaves. We also find that the effects on Chl *b* caused by *SiYGL2* mutant, natural senescence, and dark treatment are stronger than that on Chl *a* (**Figures 2A, 3A,B**). First, this may be explained by that the degradation of Chl *b* is prior to Chl *a.* And Chl *b* can converted into Chl *a* when undergoing degradation (Schelbert et al., 2009). Thus, at the preliminary stage of senescence, Chl *b* is sharply decreased while Chl *a* seems to be not impaired. With the aging process, Chl *a* is also degraded. Therefore, both the Chl *a* and Chl *b* contents are reduced in later period of senescence. Second, the reduction in Chl *b* content but not Chl *a* suggesting that the light harvesting is altered. The mutant may not grow well under low light condition, but would be fine under moderately high light. Shading and light deficiency may be one of the reasons the bottom leaf had earlier senescence. This hypothesis could be tested with a light response curve in further study.

In all leaves at different ages, both ΔΦPS II and ΔETR were lower in *siygl2* than in WT (**Figure 3D**), indicating that the PS II function was impaired in *siygl2*. Thus, we propose that SiYGL2 plays a role in the regulation of PS II function. In conclusion, in monocotyledonous *S. italica*, SiYGL2 is associated with the regulation of leaf senescence, chlorophyll metabolism, and PS II efficiency. As described in previous studies, AtEGY1 participates in chlorophyll accumulation, leaf senescence, and PS II function (Chen et al., 2005, 2016). This indicates that the function of SiYGL2 in monocotyledonous *S. italica* is quite similar to that its homolog AtEGY1 in dicotyledonous *Arabidopsis*.

### The HEXXH Motif May Be Essential for the Function of SiYGL2 in Chloroplast Development

The *Arabidopsis* mutant *egy1-1* displayed a defective chloroplast development phenotype (Chen et al., 2005). However, the *S. italica EGY1* mutant *siygl2* has relatively normal chloroplasts (**Figure 4C**). Further analysis of the function of SiYGL2 in chloroplast development is therefore required. We compared the peptide chains of these two mutants and found that the mutant AtEGY1 protein lacks the HEXXH and NPDG motifs, while the mutant SiYGL2 protein lacks only the NPDG motif. Thus, we propose two hypotheses for the differences in chloroplast development between the *egy1-1* and *siygl2* mutants. First, it has been reported that for S2P family members in *Bacillus subtilis*, HEXXH and NPDG together form the catalytic center required for protease function (Rudner et al., 1999). Furthermore, two other MPs, AtVAR2 and AtVIR3, which have the HEXXH motif but lack the NPDG motif, could regulate chloroplast development (Chen et al., 1999; Qi et al., 2016). We, therefore, speculate that HEXXH is essential for chloroplast development-related function of EGY1 and alone can guarantee normal chloroplast development, but requires NPDG to regulate leaf senescence. Second, SiYGL2 and AtEGY1 share 77.3% amino acid sequence similarity, so the differences between remaining amino acids may lead to differences in function. Homologous proteins commonly show different functions in different species; EGY1 may potentially have changed its chloroplast development-related function in dicotyledonous C_3_ *Arabidopsis* relative to the monocotyledonous C_4_ plant *S. italica* during evolution. These two hypotheses could be verified in future by removing the HEXXH motif of SiYGL2 and observing the chloroplast structure of the resulting plants.

### Chlorophyll Content and PS II Efficiency May Be Not the Limiting Factors of Photosynthesis Capacity

Chlorophylls functions as the antenna pigment in the photosynthesis and as such is an essential component to PS II function (Stirbet et al., 2014; Kato et al., 2016). However, while chlorophyll content, ΔΦPS II, and ΔETR were lower in *siygl2* than in WT (**Figures 10B,C**), net photosynthetic rate of *siygl2* remained the same as in the WT (**Figure 2B**). Similar results have been reported in the *S. italica ygl1* mutant and rice *ygl7* mutant, which are two mutants of D submit of Mg-chelatase encoding gene, that show decreased chlorophyll levels and defective chloroplasts and, yet display increased photosynthetic rates and photosynthetic reaction center activity (Deng et al., 2014; Li et al., 2016). A previous report has stated that plants contain an abundance of light-harvesting pigments and absorb more light than they can use (Ort et al., 2015). Following this, we speculate that in the *siygl2* mutant leaves, even though chlorophyll accumulation is reduced, photosynthesis can still be maintained at the same level as in the WT because sufficient light is still harvested by the chlorophyll present. The fact that a reduction in PS II efficiency does not impair the photosynthetic rate of the mutant may suggest that the *siygl2* mutant may have improvements in other photosynthesis-related components that compensate for the decrease in PS II efficiency. In addition, the induction of alternative electron transport (water–water cycle, etc.) may be also a reason leading to the alteration in ΔETR but not photosynthetic rate. These should be confirmed by further experiments.

### Accelerated Leaf Senescence and Panicle Chlorosis May Be Associated With Yield Decreases in *siygl2* Mutants

Despite the conserved photosynthetic rate, the yield of *siygl2* was significantly reduced when compared with WT plants. We propose two explanations for this phenomenon. On the one hand, yield is closely related to leaf senescence, so accelerated leaf senescence could influence the mobilization of nutrients to reproductive organs and reduce the grain yield. Alternatively, the panicle has photosynthetic carbon assimilation capabilities and plays an important role in grain formation. Glumes can ensure remobilization of nutrients to grains as a vital part of crop source–sink translocation. Compared with the flag leaf, glumes are more crucial in the later period of grain filling (Kong et al., 2015). In our study, the *siygl2* mutant panicle contained only 28% of the chlorophyll contained in the WT panicle at the heading stage (**Figure 2**). This suggests that the low chlorophyll content and impaired photosynthetic capability of the glume may be associated with the reduced yield in the *siygl2* mutant.

## Author Contributions

XD conceived the project. SZ, WL, JS, and CT carried out the experimental work. HZ provided the materials and did the field trials. SZ did the data analysis and wrote the manuscript. XD, GJ, and ST guided the experimental work. All authors read and approved the final manuscript.

## Conflict of Interest Statement

The authors declare that the research was conducted in the absence of any commercial or financial relationships that could be construed as a potential conflict of interest. The reviewer RZ and handling editor declared their shared affiliation at the time of the review.

## Footnotes

1 https://phytozome.jgi.doe.gov/

2 https://blast.ncbi.nlm.nih.gov/

3 http://www.cbs.dtu.dk/services/TMHMM/

4 http://phytozome.jgi.doe.gov/pz/portal.html

## Supplementary Material

FIGURE S1 The plastoglobulis areas of the young leaves and old leaves in Yugu1 and *siygl2*. Means and standard deviations are obtained from 10 plastoglobulis. Statistics treatment was made with Welch’s two-sample *t* test. ^∗∗^Significantly different at *P* = 0.01.

FIGURE S2 Sequences at the mutation sites of Yugu1, *siygl2, gh120*, and F_1_ individuals. The red boxes and arrow show the mutant sites.

TABLE S1 CAPS markers for fine mapping.

TABLE S2 Sequencing primers for the candidate region.

TABLE S3 Primers used for qRT-PCR analysis.
