# Supplementary figures and images for: SiYGL2 Is Involved in the Regulation of Leaf Senescence and Photosystem II Efficiency in Setaria italica (L.) P. Beauv
Source: Front Plant Sci. 2018 Sep 4;9:1308. doi: 10.3389/fpls.2018.01308 (PMC6131628; doi:10.3389/fpls.2018.01308)

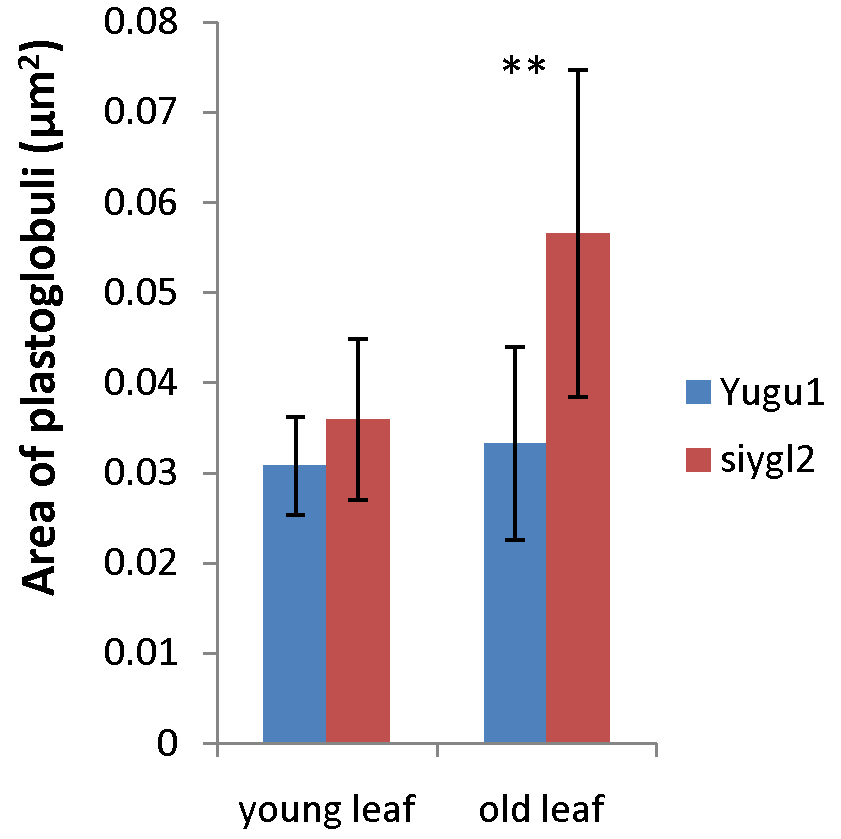

Supplement: FIGURE S1 — The plastoglobulis areas of the young leaves and old leaves in Yugu1 and siygl2. Means and standard deviations are obtained from 10 plastoglobulis. Statistics treatment was made with Welch’s two-sample t test. ∗∗Significantly different at P = 0.01. [file Image_1.TIF]

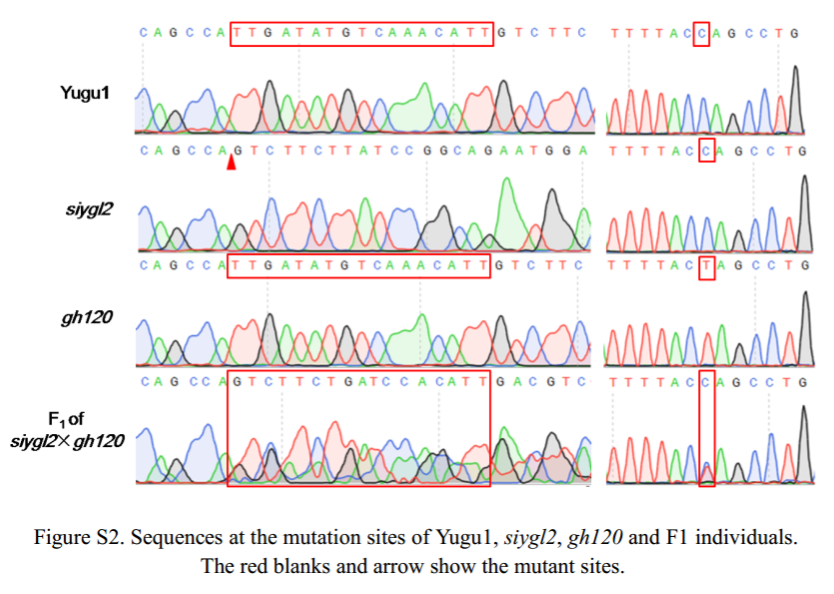

Supplement: FIGURE S2 — Sequences at the mutation sites of Yugu1, siygl2, gh120, and F1 individuals. The red boxes and arrow show the mutant sites. [file Image_2.TIF]
