# Supplementary material for: SiYGL2 Is Involved in the Regulation of Leaf Senescence and Photosystem II Efficiency in Setaria italica (L.) P. Beauv
Source: Front Plant Sci. 2018 Sep 4;9:1308. doi: 10.3389/fpls.2018.01308 (PMC6131628; doi:10.3389/fpls.2018.01308)
Supplement: TABLE S1 — CAPS markers for fine mapping. [file Table_1.DOCX]

**Table S1. CAPS markers for fine mapping**

| Primer name | Forward primer sequence | Reverse primer sequence |
| --- | --- | --- |
| ZS901 | ACCCTGGCATCTTCTT | CTTTACCCTCGGCTTG |
| ZS902 | AAACCCTTTGTGGAGT | TACCCGTTATTTGACC |
| ZS903 | AGCCATCGTTGTCTTCC | CCCGTATCCTCTTTCG |
| ZS904 | ATTCTACGGATGAAACAGC | AACAACACCGACAAGATAA |
| ZS905 | ATTTAGCAAGGGATTCAG | ATGGCAGTCCAGTAGTGTTTCA |
| ZS906 | GTCTCGCCGTCCTGTTTAT | CCAGCAAGCAGAGCCAC |
| ZS907 | TTCGTTTGGGAGGGTT | TGGCAGAGGAAGAGGC |
| ZS908 | GCAGTTATGTTAGGGTATT | ATGAGTATGTTGTGAGGC |
| ZS909 | AGGCATGGTATGGAGG | AAACGAGGAAACAGGAA |
| ZS910 | GGAAGAGTTTGGTGCTG | TTGTTCAATGATAGGTCGT |
| ZS911 | TCATACTCCCATTACTTTC | GTGTTGGCTCACTCCT |
| ZS912 | TGTCAAGAAGCCTGTTA | TGAAATCGGGATAAAG |
| ZS913 | GTCAACCAACCACCAAATAAC | ACGACGAGGATGATGAAGAA |

**Table S2. Sequencing primers for candidate region**

| Primer name | Forward primer sequence | Reverse primer sequence |
| --- | --- | --- |
| sseq019 | AGGTAGCGAGTTCTTGTC | TTGGGATTCAGTTGGA |
| sseq020 | TAACACCCTCCCTCAA | CCTTCACAAGCACCTC |
| sseq021 | CATGAAGATATGTGGCAGTA | GGAGATCGTCCCTAACC |
| sseq022 | ACGGTGCGGGTCATAT | CACTGCGGTTCAACTACTC |
| sseq023 | CTGGGCTCGCTGTACTTG | CCTTTCCCTTCCTTTGC |
| sseq024 | GAGTCGTCGCAATCAGGC | CAGGGTTTGGCAGGTTC |
| sseq025 | CACATGCGTCGTCCAG | ACCACCCAACACCTCC |
| sseq026 | CCGCTTCAGTAATCAGG | TACGCAGGCACAAACA |
| sseq027 | AAACTCCCTCTGTATTGC | CGTTGGGTCTTCTGGTA |
| sseq028 | TCCTCCTTTCATCTGGG | GGCTTCACTTCATTACGG |
| sseq029 | TTTGGTTGGCATCTCC | CTGCTTTCTTGGGATTAG |
| sseq030 | CCAAATGGCACTTAACTCG | TCACCGTAGCACAGGACA |
| sseq031 | TGGAGCAGTGACTACAGTGTTT | TGGTATCAATGTGACGGTTTA |
| sseq032 | CCGAGGAAGGTTAGGC | GGTGCGTTTCAGGTTT |
| sseq033 | GTCGTCGTCATCCAGG | TGTTCGGTTTGTTTCG |
| sseq034 | ATAGATAGGGCTCCACTG | TTTGTTGCGATTCTGC |
| sseq035 | CAACCAACCACCAAATAACG | GGTCAGGATTAGAAACCGAAA |
| sseq036 | GCGGACGCTATCACTG | CAAATGCTACATCGGAGA |
| sseq037 | CATGAAGATATGTGGCAGTA | GGAGATCGTCCCTAACC |
| sseq038 | CCTCCTATGCTTCTGCC | CACTGCGGTTCAACTACTC |
| sseq039 | CTTCATGCCGTTCTTGC | ATCGGATGTTTGATGCTAAT |
| sseq040 | AGCGAATGCCTCTTCTTC | GCTTGCCGTAGGTCTGC |
| sseq041 | GTCGTCGCAATCAGGC | CAGGGTTTGGCAGGTTC |
| sseq042 | CACATGCGTCGTCCAG | ACCACCCAACACCTCC |
| sseq043 | CTCCGAGCTAGATCCACT | TACGCAGGCACAAACA |
| sseq044 | AAAGAGCGAACCTGTGA | AATAAACGGCGGGAAG |
| sseq045 | CATCCTGGCTCTAGTTGT | CACTTCCTTGGGACTTT |
| sseq046 | GGACGCTATCACTGCC | CACAATGCTGGAAACG |
| sseq047 | CGCTGCTTCTCGGTTTC | GCTTGCCGTAGGTCTGC |
| sseq048 | GAGTCGTCGCAATCAGGC | TCAGGGTTTGGCAGGTTC |
| sseq049 | AGAACCTGCCAAACCCTG | TGACCACCCAACACCTCC |
| sseq050 | AAAGAGCGAACCTGTGA | GCCTAAAGCGACGAAG |
| sseq051 | TGTTCTCCTCCATTCTGT | CTGCTTTCTTGGGATTAG |
| sseq052 | AGATAGGGCTCCACTG | GCTGTTTGTTGCGATT |
| sseq053 | CGACGAGCACGAGCAT | CCCAATCCGATTCAATAGTT |
| sseq054 | GGCTCAGTACGAACCAGTC | AATCGGAAGCACAAGAATT |
| sseq055 | GCACTTCGTCGCTTTA | TTGTTCAATGATAGGTCGT |
| sseq056 | AGATAGGGCTCCACTG | AACGAAATGCGAATAG |
| sseq017 | GAACTTGCGGAGGACC | GCTCACAGCCCAAATGA |
| sseq018 | ATCAACGCTGCGAGAC | GTACGCCGAAAGAACC |
| sseq052 | AGATAGGGCTCCACTG | GCTGTTTGTTGCGATT |
| sseq053 | CGACGAGCACGAGCAT | CCCAATCCGATTCAATAGTT |
| sseq054 | GGCTCAGTACGAACCAGTC | AATCGGAAGCACAAGAATT |
| sseq055 | GCACTTCGTCGCTTTA | TTGTTCAATGATAGGTCGT |
| sseq056 | AGATAGGGCTCCACTG | AACGAAATGCGAATAG |
| sseq017 | GAACTTGCGGAGGACC | GCTCACAGCCCAAATGA |
| sseq018 | ATCAACGCTGCGAGAC | GTACGCCGAAAGAACC |

**Table S3. Primers used for qRT-PCR**

| Primer name | Forward primer sequence | Reverse primer sequence |
| --- | --- | --- |
| SiYGL2 | ATATCAATGGCTGGTCCTGTGG | GCAGCAAGGAACCCTGGAATAA |
| SiNYC1 | GGCATTCCCACTTCTGTAAGCTA | TACATGGTGCAGGCTACTTCC |
| SiSAG12 | TGCTGTCGAGGGAATGAACC | TGGTAGCCGTTGATCGTGAC |
| SiPAO | CGTGCTCCCGCATATCTTCC | CACCGCCGAGAAGTGAACAA |
| SiSGR1 | AGTGAGACTGCATGTACGGG | TTCCCTGACCTCAACCAAGC |
| SiRCCR | CTGTCCCGTGTTCCACTGAA | AGATGCAAGGGCTCTGTTCC |
